# Association of pulse wave velocity and intima‐media thickness with cardiovascular risk factors in young adults

**DOI:** 10.1111/jch.13812

**Published:** 2020-01-19

**Authors:** Marina Cecelja, Raja Sriswan, Bharati Kulkarni, Sanjay Kinra, Dorothea Nitsch

**Affiliations:** ^1^ Cardiovascular Division Department of Clinical Pharmacology King's College London British Heart Foundation Centre St Thomas' Hospital London UK; ^2^ London School of Hygiene and Tropical Medicine London UK; ^3^ National Institute of Nutrition Hyderabad India

**Keywords:** arterial stiffness, atherosclerosis, intima‐media thickness, pulse wave velocity, risk factors

## Abstract

Pulse wave velocity (PWV), a measure of arterial stiffness, and intima‐media thickening (IMT), a measure of early atherosclerosis, are intermediate markers of cardiovascular disease which are predictive of cardiovascular events. Traditionally, both were thought to result from accumulative exposure to traditional cardiovascular risk factors. However, their association with risk factors in young adults in low‐income settings is unknown. We sought to investigate the association between PWV and IMT with traditional cardiovascular risk factors in the Andhra Pradesh Children and Parents Study cohort from Southern India. Male and female adults (N = 1440) aged between 20 and 24 years underwent measures of PWV and IMT. Exposure variables included smoking, body mass index (BMI), mean arterial pressure (MAP), glucose, homeostatic model assessment of insulin resistance (HOMA‐IR), total cholesterol, high‐density lipoprotein cholesterol (HDL‐cholesterol), and triglycerides. Association between outcome and exposure variables was assessed using linear regression analysis. Average values for PWV and IMT were 5.9 ± 0.6 m/s and 0.5 ± 0.1 mm. In univariable analysis, PWV associated with MAP, BMI, smoking, total cholesterol, glucose, and HOMA‐IR and IMT associated with MAP, BMI, tobacco use, and HDL‐cholesterol. In multivariable analysis, PWV remained strongly positively associated with MAP increasing by 0.5 m/s (*P* < .001) for a 10 mm Hg increase in MAP (*R* ^2^ = .37). In contrast, IMT negatively associated with HDL‐cholesterol (*β* = −.10; *P* = .012, *R* ^2^ = .02). There was weak evidence that PWV and IMT positively associated with BMI. In young adults from Southern India, PWV positively associated with blood pressure and IMT negatively associated with HDL‐cholesterol. This suggests separate etiologies for atherosclerosis and arterial stiffening in young adults.

## 1 INTRODUCTION

Noncommunicable disease accounts for two‐third of deaths worldwide, of which 80% occur in low or middle‐income countries.^1^ Cardiovascular disease is a leading cause of morbidity and mortality from noncommunicable disease. Two main pathologies underlying cardiovascular disease are (a) development of atherosclerosis and (b) vascular remodeling which is often concomitant with atherosclerosis but can occur independently.^2^ While western countries have seen a recent reduction in age‐adjusted cardiovascular mortality rates,^1^ mortality rates have continued to rise in low‐income countries.^3^ In particular, India has some of the highest cardiovascular mortality rates in the world reaching an estimate of 349 deaths per 100 000 in men and 265 per 100 000 in women.^3^ This rise in cardiovascular mortality is closely associated with demographic shifts, epidemiological transition, and urbanization leading to changing behavior and dietary patterns.

Cardiovascular disease is a chronic disease which develops as a result of exposure to risk factors from childhood and throughout the life course.^4^ Structural and functional changes that occur in large arteries with age and from exposure to cardiovascular risk factors are important intermediate markers of subclinical cardiovascular disease. Of particular interest are measures of intima‐media thickness (IMT) and pulse wave velocity (PWV). These measurements relate to development of atherosclerosis and vascular remodeling, respectively. In addition, both PWV^5^ and IMT^6^ are predictors of cardiovascular morbidity and mortality and strategies aimed at reducing their progression in early adulthood could reduce premature cardiovascular morbidity and mortality.

Traditionally, both IMT and PWV were thought to result from accumulative exposure to cardiovascular risk factors over the life course, explaining their association with cardiovascular morbidity and mortality.^7^ While an association between IMT and cardiovascular risk factors is well established,^8^ observational data suggest that PWV is primarily influenced by increased blood pressure (BP) and diabetes^9^ but not other cardiovascular risk factors. However, these findings are based on data from high‐income countries and across a wide range of age groups. A little is known about the association between IMT and PWV to traditional cardiovascular risk factors in low‐ and middle‐income countries and at the early stages of vascular pathologies. To date, most of the studies looking at the association between PWV and traditional risk factors in young adults have been conducted in high‐income countries.^10^, ^11^, ^12^, ^13^, ^14^, ^15^, ^16^, ^17^, ^18^ Two studies have been conducted in South Africa^19^ and Brazil^20^ but with a relatively small sample size of <220 participants. There are currently no studies looking at the association between both PWV and IMT to traditional risk factors in young individuals in low‐ or middle‐income countries, including South‐Asian communities.

The aim of the present study was to investigate the cross‐sectional association between PWV and IMT with traditional cardiovascular risk factors in male and female young adults aged between 20 and 24 years in the Andhra Pradesh Children and Parents Study (APCAPS) cohort conducted in Southern India.

## 2 METHODS

### 2.1 Cohort profile

**Figure 1:**
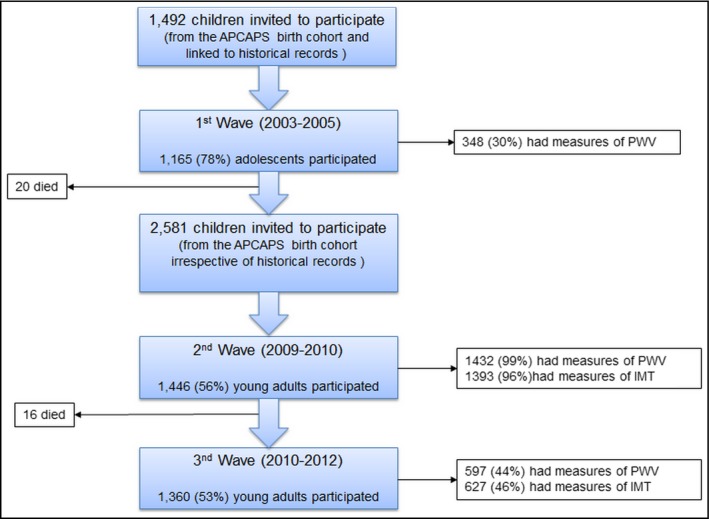
Flow diagram of the number of participants adapted from Kinra et al^21^

Andhra Pradesh Children and Parents Study is built upon the Hyderabad Nutrition Trial (HNT), Southern India, conducted between 1987 and 1990. APCAPS is a prospective cohort study which aimed to follow‐up the children that took part in the HNT trial as previously described and is outlined in Figure 1.^21^ For the present study, data collected during the second wave between 2009 and 2010 was used in the analysis. The APCAPS study was approved by the Ethics Committee of the National Institute of Nutrition, Hyderabad, India, University of Bristol and the London School of Hygiene and Tropical Medicine, UK. Written informed consent was obtained from all subjects. The present analysis was approved by the London School of Hygiene and Tropical Medicine Ethics Committee. The study protocol conforms to the ethical guidelines of the 1975 Declaration of Helsinki.

### 2.2 Outcome variables

**Figure 2:**
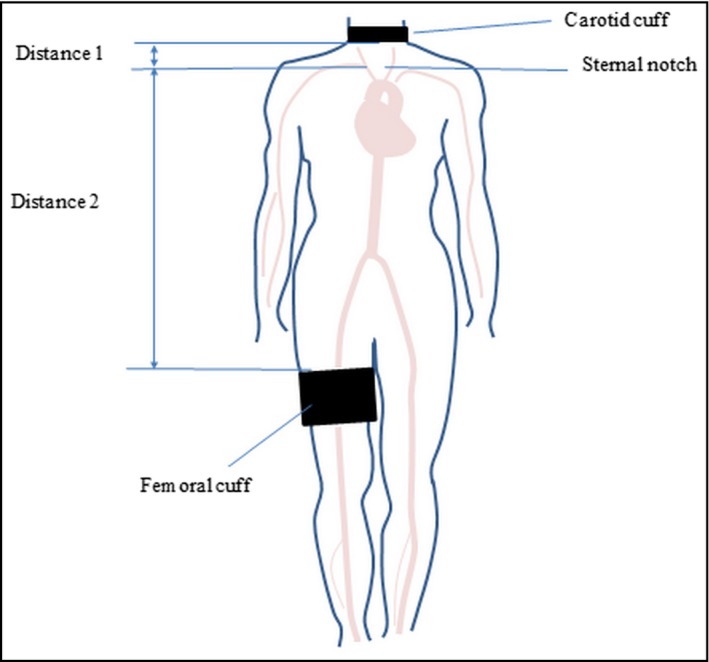
Carotid cuff location around the neck and femoral artery cuff around the upper thigh and distance measurements adapted from Bailey et al^43^

Pulse wave velocity was measured using the Vicorder device (Skidmore Medical Limited).^21^ Carotid and femoral artery pressure waveforms were recorded simultaneously by placing BP cuffs around the neck (30 mm wide cuff) and upper thigh (100 mm wide cuff) with the subjects in the supine position (Figure 2). The cuffs were inflated to 60 mm Hg, and pressure waveforms were recorded for 3 seconds using a volume displacement method. The foot of the pressure waveform was identified using a cross‐correlation algorithm centered at the peak of the second derivative of pressure. The difference in time between pulse arrival at the carotid artery in comparison with the femoral artery was taken as the transit time. The difference in distance between the two sites was measured using a tape measure from the upper edge of the femoral cuff (distance 2, Figure 2) to the sternal notch minus the distance between the lower edge of the carotid cuff to the sternal notch (distance 1, Figure 2). PWV was calculated by dividing the distance by the transit time in meters per second (m/s). PWV was measured three times, and the average was used for all further analysis.

For measurement of IMT, carotid artery was visualized using B‐mode ultrasonography (Ethiroli Tiny‐16a, Surabhi Biomedical Instrumentation India). IMT was measured as the distance between the first and second bright lines which represent the lumen‐intima interface and media‐adventitia interface, respectively. This technique has been shown to relate closely to measurements made using pathological evaluations^22^ with a mean difference of 0.02‐0.14 mm between repeat measures.^23^ IMT was measured offline using an image analysis package (Carotid Plaque Texture Analysis Software, Copyright LifeQ Ltd). Two measurements of IMT were made by separate operators and averaged, and the mean value of IMT was used for further analysis. Previous studies have reported good reproducibility of IMT measurement using ultrasound with an intra‐ and inter‐observer variability of 5.4%‐5.8% and 10.5%, respectively.^24^

### 2.3 Exposure variables

#### 2.3.1 Blood pressure and heart rate

Blood pressure was measured three times, and an average was used for analysis. Measurements were made using an oscillometric device Omron HEM 7300, which has been validated for accuracy according to the British Hypertension Society guidelines. MAP was calculated as [(2 × DBP) + systolic]/3. Heart rate (HR) was measured in triplicate using Omron M5‐I (Omron, Matsusaka Co.), and an average of the three measurements was used for analysis. HR was normally distributed.

#### 2.3.2 Smoking

Information on whether a participant had smoked, chewed, or snuffed tobacco on a regular basis (at least weekly) was gathered as part of a questionnaire. Each participant was categorized according to the status of smoking, chewing, or snuffing tobacco as: current (used in the last 6 months), former (ceased use >6 months ago), or never smoked, chewed, or snuffed tobacco.

#### 2.3.3 Blood lipids

Total cholesterol was measured using an enzymatic cholesterol oxidase method, HDLcholesterol was measured using an homogeneous assay, and triglycerides were measured using enzymatic colorimetric, GPO‐PAP method (Instrument: Synchron CX9, Reagent source: BeckmanCoulter). LDL‐cholesterol was not used in analysis as it is derived from total cholesterol and HDL‐cholesterol and would introduce multicollinearity in the data analysis (observed correlation between LDL and total cholesterol was *r* = .94).

#### 2.3.4 Body mass index

For calculation of participant's BMI, weight (kg) measurements were made in duplicate using a digital weighing machine (Model PS16, Beurer). Height (mm) was also measured in duplicate with the participant standing using a portable Seca Leicester height measure, Chasmors. BMI was then calculated using the formula weight (kg)/height^2^ (m^2^), where weight and height were averages of the duplicate measures.

#### 2.3.5 Glucose and insulin

Glucose was measured by an enzymatic method using glucose oxidase/peroxidase‐4‐aminophenazone‐phenol method. Insulin was measured by an enzyme‐linked immunosorbent assay method using solid phase, two‐site enzyme immunoassay (Instrument: Rayto, Reagent source: Mercodia). Insulin resistance was calculated by multiplying measures of fasting insulin with fasting glucose to give the homeostatic model assessment of insulin resistance (HOMA‐IR) which was used for further analysis.

#### 2.3.6 Alcohol consumption

Information on alcohol consumption was gathered as part of a questionnaire. Alcohol consumption was split according to intake of local spirits, branded spirits, wine, and beer and frequency of consumption recorded. For each type of alcohol frequency of present consumption was classified according to the following categories: daily/most days, weekends only, 1‐2 times a month, special occasions, and never. For further analysis, the alcohol type with the highest frequency of drinking was used.

### 2.4 Data analysis

Data analysis was performed using Stata version 14. A small number of individuals had a history of stroke (n = 1), coronary heart disease (n = 3) and taking medication for diabetes (n = 2), these participants were removed from further analysis giving a total sample size of 1440 participants (Table 1). For PWV, out of 1440 participants, 1426 had measures of PWV. Of these 1400 had PWV and no missing values for the other exposure variables and were included in the final multivariable model. For IMT, out of 1440 participants 1389 had measures of IMT. Of these, 1362 had measures of IMT and no missing values for the exposure variables and were included in the final multivariable model. Subject characteristics are presented as mean and standard deviation for continuous variables and number and percentage for categorical variables. Comparison between variables by sexand between those with complete and those with missing data was performed using Student's *t* test for continuous variables, chi‐square test for binary variables and chi‐square test for trend for ordered categorical variables.

**Table 1:** Characteristics of all participants and bysex

Characteristics (units, n = number of observations)	Total cohort (N = 1440)	Male (N = 858)	Female (N = 575)	*P*‐value
Age (y, n = 1440)	20.9 ± 1.6	20.8 ± 1.1	20.9 ± 1.2	.017
BMI (kg/m^2^, n = 1439)	19.5 ± 2.8	19.5 ± 2.8	19.5 ± 3.0	.770
MAP (mm Hg, n = 1424)	84.0 ± 7.5	84.2 ± 7.5	83.7 ± 7.5	.297
Tobacco^a^ (n = 1438)				
Never (n, [%])	1239 [86.2]	715 [83.4]	519 [90.3]	<.001^b^
Former (n, [%])	12 [0.8]	11 [1.3]	1 [0.2]	
Current (n, [%])	187 [13.0]	131 [15.3]	55 [9.6]	
Alcohol^c^ (n = 1438)				
Daily/most days (n, [%])	30 [2.1]	22 [2.6]	8 [1.4]	<.001^b^
Weekends only (n, [%])	194 [13.5]	145 [16.9]	49 [8.5]	
1‐2 times a month (n, [%])	277 [19.3]	207 [24.2]	67 [11.6]	
Special occasions (n, [%])	234 [16.3]	153 [17.9]	81 [14.1]	
Never (n, [%])	703 [48.9]	330 [38.5]	370 [64.4]	
Total cholesterol (mg/dL, n = 1431)	154.1 ± 34.5	153.4 ± 33.6	155.2 ± 35.9	.330
HDL‐cholesterol (mg/dL, n = 1431)	39.5 ± 7.6	38.8 ± 7.3	40.5 ± 7.8	<.001
Triglycerides (mg/dL, n = 1431)	101.7 ± 51.7	104.5 ± 57.0	97.5 ± 42.0	.012
Glucose (mg/dL, n = 1431)	86.4 ± 9.6	86.5 ± 9.6	86.3 ± 9.4	.687
Insulin (Uu/mL, n = 1431)	4.7 ± 3.7	4.6 ± 3.0	5.0 ± 4.4	.056
PWV (m/s, n = 1419)	5.9 ± 0.6	5.9 ± 0.6	5.8 ± 0.6	.091
IMT (mm, n = 1382)	0.54 ± 0.12	0.55 ± 0.16	0.54 ± 0.13	.145

Abbreviations: BMI, body mass index; DBP, diastolic blood pressure; HDL, high‐density lipoprotein; HR, heart rate; IMT, intima‐media thickness; MAP, mean arterial blood pressure; PWV, pulse wave velocity; SBP, systolic blood pressure.

a Smoking or chewing tobacco, former user = ceased use >6 mo ago; current user = used in the last 6 mo.

b Chi‐ squared test for trend.

c Highest frequency of local spirits, branded spirit, wine or beer.

Univariable linear regression analysis was used to assess the association of PWV and IMT (outcome variables) to cardiovascular risk factor (exposure variable) adjusting for village clustering using robust standard errors using the cluster function within Stata, age, and sex. The number of individuals included in analysis, beta coefficient, 95% confidence interval, *P*‐values (obtained using the Wald test), and coefficient of determination (*R* ^2^) are presented in the results. Because the final multivariable model included only individuals without any missing data, univariable analysis was repeated only in individuals with complete data to ensure findings were consistent (Table S1 and S2). Linear regression assumes that residuals are normally distributed, and the validity of this assumption was checked using a normal quantile plot. The assumption that there is constant variance among dependent and independent variables was checked by plotting standardized residuals against the fitted values. In this analysis, it is assumed that the relationship between the outcome and exposure is linear. Linearity of an association was examined by adding quadratic terms to the model and significance examined using the Wald test. For easier interpretability of the beta coefficients, IMT was scaled up by a factor of ten and total cholesterol, triglyceride, serum glucose, HDL‐cholesterol, and MAP were divided by 10.

For PWV, multivariable analysis model building initially included MAP and measures of impaired glycemia as these were hypothesized to be associated with PWV and were entered into the model first, adjusting for age, sex, and village cluster. Secondly, the model additionally tested for interactions between HR and MAP. The final model additionally included all other risk factors to investigate whether these were associated with PWV in this population. For IMT, model building initially included risk factors mainly associated with development of atherosclerosis including smoking, obesity, and dyslipidemia. Secondly, alcohol intake was included in the model and any confounding effect on smoking was examined. The final model additionally included all other risk factors to investigate whether these were associated with IMT. For easier interpretability, only variables with a *P* value <.10 and those hypothesized to be associated with the outcome variables are presented in the final model. Standard errors were examined for each new variable for evidence of collinearity. Standardized beta regression coefficient, 95% CI, *P*‐values and *R* ^2^ coefficient from the final model are presented in the results section. Linear regression assumptions for the final regression model were assessed as described for univariable analysis.

## 3 RESULTS

### 3.1 Participant characteristics

The mean age of all participants was 20.9 ± 1.6 years and 60% of participants were male (Table 1). Average values for IMT and PWV were 0.5 ± 0.1 mm and 5.9 ± 0.6 m/s, respectively, with weak evidence that PWV was slightly higher in men compared to women (mean PWV 5.9 vs 5.8 m/s, *P* = .09). Average BMI was 19.5 ± 2.8 kg/m^2^. Most participants had never used tobacco (86%), 13% were current smokers and 1% were former smokers with a higher prevalence of current male smokers compared to women (15% vs 10%). Forty‐nine percent of participants had never had alcohol and most individual that did drink did so 1‐2 times a month (19%) or on special occasions (16%). Fourteen percent drank on weekends and only 2% drank on a daily basis. More men drank alcohol compared to women (61% vs 36%). Average values for total cholesterol, HDL‐cholesterol and triglycerides were 154 ± 35, 40 ± 8 and 102 ± 52 mg/dL, respectively. Average values for glucose and insulin were 86 ± 10 mg/dL and 4.7 ± 4 Uu/mL, respectively. Men had higher triglycerides (105 vs 98 mg/dL), lower insulin (4.6 vs 5.0 Uu/mL), and lower HR (73 vs 80 bpm) when compared to women.

### 3.2 Univariable analysis

After adjusting for age, sex, and village clusters, there was strong evidence of a positive linear association between PWV and MAP, BMI, total serum cholesterol, triglycerides, glucose, and HOMA‐IR (Table 2). There was no association with HDL‐cholesterol. There was strong evidence of a negative association between PWV and frequency of drinking and weak evidence of an association with smoking status (*P* = .057).

**Table 2:** Univariate linear regression analysis of the association of PWV and IMT (per 10 mm) to cardiovascular risk factors adjusted for age, sex, and village level clustering (analysis is limited to participants with complete data with each line representing separate models)

Variable	Pulse wave velocity			Intima‐media thickness		
	*β*	95% CI	*P*‐value	*β*	95% CI	*P*‐value
MAP (per 10 mm Hg)	.48	0.43, 0.52	<.001	.04	−0.07, 0.16	.453
BMI (kg/m^2^)	.06	0.04, 0.07	<.001	.02	0.00, 0.04	.065
Tobacco categories^a^						
Never	Reference		.057^b^	Reference		.017^b^
Former	.25	−0.18, 0.69		−.29	−0.07, 0.18	
Current	.14	−0.02, 0.29		.25	0.02, 0.49	
Alcohol category^c^						
Daily/most days	Reference		<.001^b^	Reference		.032^b^
Weekends only	.17	−0.06, 0.40		−.12	−0.69, 0.44	
1‐2 times a month	.15	−0.09, 0.37		−.24	−0.97, 0.49	
Special occasions	.16	−0.09, 0.41		−.21	−0.97, 0.55	
Never	.03	−0.19, 0.25		−.39	−1.15, 0.38	
Total cholesterol (per 10 mg/dL))	.03	0.01, 0.04	<.001	.00	−0.02, 0.01	.529
HDL‐cholesterol (per 10 mg/dL)	.02	−0.03, 0.08	.335	−.10	−0.18, −0.03	.007
Triglycerides (mg/dL)	.24	0.13, 0.35	<.001	−.01	−0.13, 0.11	.828
Glucose (per 10 mg/dL)	.06	0.03, 0.09	<.001	−.05	−0.12, 0.03	.207
HOMA‐IR	.17	0.11, 0.23	<.001	−.08	−0.19, 0.02	.116

Note

Analysis was adjusted for age, sex, and village clustering.

Abbreviations: BMI, body mass index; HDL, high‐density lipoprotein; HOMA‐IR, homeostatic model assessment of insulin resistance; HR, heart rate; MAP, mean arterial blood pressure; PWV, pulse wave velocity.

a Smoking or chewing tobacco, former user = ceased use >6 mo ago; current user = used in the last 6 mo.

b *P*‐value for all categories.

c The highest frequency for either local spirits, branded spirit, wine or beer.

There was evidence of a positive association between IMT and tobacco use (*P* = .017), frequency of drinking (*P* = .03), BMI (*P* = .065) and a negative association with HDL‐cholesterol. There was no evidence of an association between IMT and MAP, total cholesterol, triglyceride, and glucose.

### 3.3 Multivariable analysis

The first model included PWV as the outcome variable and MAP, glucose and HOMA‐IR as explanatory variables, adjusting for age, sex, and village clusters only. MAP remained strongly associated with PWV, similarly to univariable analysis PWV increased by 0.5 m/s (95% CI: 0.43, 0.51; *P* < .001, Table 3) for an 10 mm Hg increase in MAP. There was no evidence of an association between PWV and glucose or HOMA‐IR. The second model additionally included an interaction term between HR and MAP. There was no evidence of an association between PWV and HR or evidence that HR modified the association between PWV and MAP. The final model additionally adjusted for all other cardiovascular risk factors. The results remained similar for the association between PWV and MAP, glucose, and HOMA‐IR and the size of the association between PWV and MAP was very slightly affected with the beta coefficient moving a little toward zero showing minimal confounding by other risk factors. There was evidence of an independent positive association between PWV and BMI. Inclusion of BMI increased the percentage variance explained by the multivariable model from 37% to 38%. Recalculated MAP using only the last 2 blood pressure measurements as recommended by the European guidelines for hypertension^25^ did not change the strength of the association with pulse wave velocity. There was no evidence of an independent association between PWV with HR, total cholesterol, HDL‐cholesterol, triglycerides, smoking or alcohol. The results remained unchanged in sensitivity analysis that excluded outliers (Table S3).

**Table 3:** Final multivariate regression model of the association between PWV (m/s) and cardiovascular risk factors

	*β*	95% Confidence interval	*P*‐value	*R* ^2^
Model 1 (adjusted for age,sex, and village clusters)				
MAP (per 10 mm Hg)	.47	0.43, 0.51	.001	.37
Glucose (per 10 mg/dL)	.01	−0.02, 0.05	.385	
HOMA‐IR	.00	−0.04, 0.05	.840	
Model 2 (adjusted for age, sex, and village clusters, interaction term between HR and MAP)				
MAP (per 10 mm Hg)	.42	0.26, 0.58	<.001	.37
Glucose (per 10 mg/dL)	.01	−0.02, 0.05	.387	
HOMA‐IR	−.01	−0.02, 0.01	.878	
Model 3 (adjusted for age, sex, village clusters, total cholesterol, HDL‐cholesterol, triglycerides, smoking, and alcohol)				
MAP (per 10 mm Hg)	.45	0.41, 0.49	<.001	.38
Glucose (per 10 mg/dL)	.02	−0.02, 0.05	.344	
HOMA‐IR	−.01	−0.06, 0.04	.683	
BMI (kg/m^2^)	.01	−0.01, 0.02	.044	

Abbreviations: BMI, body mass index; HOMA‐IR, homeostatic model assessment of insulin resistance; MAP, mean arterial pressure; *β*, beta coefficient.

The first model included IMT as the outcome variable and total cholesterol, HDL‐cholesterol, BMI, triglycerides and smoking as explanatory variables, adjusting for age, sex, and village clusters only. Within this model, IMT remained negatively associated with HDL‐cholesterol (*β* = −.10; 95% CI: −0.01, −0.01, *P* = .013, Table 4). The beta coefficient was the same as that reported in univariable analysis suggesting that addition of other risk factors did not confound the association between IMT and HDL‐cholesterol. There was weak evidence of an independent positive association between IMT and BMI where IMT increased by 0.002 mm (95% CI: 0.00, 0.005; *P* = .098) for a one unit increase in BMI (Table 4). The beta coefficient was the same as observed in univariable analysis making confounding unlikely. There was no evidence that IMT was associated with total cholesterol, tobacco use or triglyceride levels within the model. The second model additional included alcohol use. There was no evidence of an association between alcohol and IMT but the non‐significant beta coefficients between smoking and IMT was reduced. The final multivariable model included all cardiovascular risk factors. Evidence of an independent negative association between IMT and HDL‐cholesterol and BMI remained unchanged. However, the final model accounted for only 2% of the variance in IMT. There was no evidence of an association between IMT and smoking, alcohol, BMI, glucose, HOMA‐IR, MAP, triglycerides or total cholesterol. Recalculated MAP using only the last 2 blood pressure measurements did not change the association with intima‐media thickness. The results remained unchanged in sensitivity analysis that excluded outliers and when analysis was limited to participants with IMT under 0.90 mm (Table S4 and S5, respectively).

**Table 4:** Final multivariable regression model of the association between IMT (per 10 mm) and cardiovascular risk factors

	*β*	95% Confidence interval	*P*‐value	R^2^
Model 1 (adjusted for age, sex, and village cluster)				
Total cholesterol (per 10 mg/dL)	.00	−0.01, 0.02	.663	.02
HDL‐cholesterol (per 10 mg/dL)	−.10	−0.18, −0.01	.013	
BMI (kg/m^2^)	.02	0.00, 0.05	.098	
Tobacco use^a^				
Never	Ref		.121^b^	
Former	−.23	−0.67, 0.22		
Current	.24	0.00, 0.48		
Triglycerides (per 10 mg/dL)	−.04	−0.23, 0.14	.637	
Model 2 (adjusted for age, sex, village cluster, and alcohol)				
Total cholesterol (per 10 mg/dL)	.00	−0.02, 0.02	.746	.02
HDL‐cholesterol (per 10 mg/dL)	−.10	−0.17, −0.02	.016	
BMI (kg/m^2^)	.02	0.00, 0.04	.105	
Tobacco use^a^			.216^b^	
Never	Ref			
Former	−.26	−0.72, 0.20		
Current	.15	−0.04, 0.34	‐	
Triglycerides (per 10 mg/dL)	−.06	−0.25, 0.14	.563	
Model 3 (adjusted for age, sex, alcohol, mean arterial pressure, glucose, HOMA‐IR, and village cluster)				
Total cholesterol (per 10 mg/dL)	.00	−0.02, 0.02	.686	.02
HDL‐cholesterol (per 10 mg/dL)	−.10	−0.18, 0.02	.012	
BMI (kg/m^2^)	.02	0.00, 0.05	.088	
Tobacco use^a^			.220^b^	
Never	Ref			
Former	−.27	−0.75, 0.20		
Current	.15	−0.05, 0.34		
Triglycerides (per 10 mg/dL)	−.05	−0.24, 0.15	.622	

Abbreviations: BMI, body mass index; HDL, high‐density lipoprotein; HOMA‐IR, homeostatic model assessment of insulin resistance.

a Smoking or chewing tobacco, former user = ceased use >6 mo ago; current user = used in the last 6 mo.

b *P*‐value for all categories.

## 4 DISCUSSION

The present study investigated the association between PWV and IMT to cardiovascular risk factors in young adults from a transitioning rural community in Southern India taking part in the APCAPS study. The first major finding is that PWV strongly positively associated with MAP, accounting for 37% of the variability in PWV, but not to other traditional cardiovascular risk factors. There was a modest positive association between PWV and BMI but not with other risk factors including serum cholesterol levels, lipoproteins, triglycerides, tobacco use or impaired glycemia. The second key finding in the present study is that there was a strong negative association between IMT and HDL‐cholesterol, accounting for 2% of the variability in IMT, but not with other modifiable cardiovascular risk factors.

The average PWV in the present cohort was 5.9 ± 0.6 m/s which is comparable to that observed previously for the same age group in studies conducted in high‐income countries.^10^, ^11^, ^12^, ^13^, ^14^, ^15^, ^16^, ^17^, ^18^ Our findings of an association between PWV and BP are also consistent with previous studies in young adults in high‐income countries^10^, ^11^, ^12^, ^13^, ^14^, ^15^, ^16^, ^17^, ^18^ and in one study conducted in Brazil.^20^ This suggests that the process of arterial stiffening in young individuals may be comparable between low‐ and high‐income countries. High BP may directly increase arterial stiffness by transferring stress from more compliant elastin fibers to stiffer collagen fibers at higher pressure.^26^ Alternatively and/or concomitantly, increased large artery stiffness can causally increase BP, through reduced buffering of the pulse pressure increase following left ventricular contraction.^27^ Due to the cross‐sectional nature of the present study, the direction of the relationship between PWV and BP could not be ascertained. However, a recent longitudinal study conducted in over 4000 Chinese adults showed elevated PWV to precede development of isolated systolic hypertension after adjustment for baseline BP.^28^ The present study also found a modest positive association between PWV and BMI. This association has been previously observed in cross‐sectional^11^, ^12^, ^13^, ^14^ and longitudinal studies.^29^ However, PWV measurements may be biased when distance measurements are measured over body surface area as any abdominal obesity may overestimate the distance or vice versa. In support of this, a recent study found a positive association between BMI when distance was measured using surface measurement. However, this association disappeared once aortic distance was measured using magnetic resonance imaging (MRI).^30^

Lack of association between PWV and measures of impaired glycemia in multivariable analysis is surprising as previous studies in adults have reported an association between PWV and diabetes.^9^ However, in these studies the reported association was relatively weak and accounted for 5% of the variability in PWV which may not be detectable in this young cohort of healthy individuals with low levels of glucose.^9^ In support of this, cross‐sectional analysis of young adults with type I diabetes or those classified as pre‐diabetic did not have elevated PWV compared to healthy individuals.^13^, ^14^ The lack of association between PWV and other traditional cardiovascular risk factors including smoking and measures of dyslipidemia is consistent with findings from cross‐sectional^9^ and longitudinal studies^31^ conducted in adults. Taken together this suggests that the process of arterial stiffening is separate from the pathological process of atherosclerosis and is consistent with a lack of correlation between measures of atherosclerosis and stiffening measured along the same vascular region.^32^, ^33^

The second key finding in the present study is that there was a strong negative association between IMT and HDL‐cholesterol, although this accounted for a small proportion of the variability in IMT (2%). This is consistent with some studies from high‐income countries in young adults,^34^, ^35^ but not all.^17^, ^36^, ^37^ A meta‐analysis that included >21 000 adults reported a negative association between HDL‐cholesterol and IMT, after adjustment for other atherosclerosis risk factors.^38^ Furthermore this association was maintained even at low LDL‐cholesterol suggesting a protective mechanism which is independent from LDL‐cholesterol and total cholesterol.^38^ This is consistent with the findings of the present study where we found no association between IMT and total cholesterol. Together these findings suggest a protective role of HDL‐cholesterol in IMT progression in young adults in both high‐ and low‐income countries. The mechanism by which HDL‐cholesterol may have protective effects on IMT progression is incompletely understood but is likely to involve protecting the blood vessel wall from initiation of fatty streaks by removing cholesterol from macrophages, preventing foam cell formation and reducing inflammation.^39^

A lack of association between triglyceride and IMT observed in the present study is consistent with the findings from previous studies in the same age group from high‐income countries.^17^, ^34^, ^35^, ^37^, ^40^ In addition, the present study found no association between IMT and either BMI or tobacco use. In the present study, the range of BMI was 16.7‐22.9. Previous studies from high‐income countries where BMI was associated with IMT had larger BMI range with a mean of 26.8 in the Muscatine offspring study,^36^ 24.7 in the ARYA study^17^ and 28.0 In the Bogalusa Heart study.^35^ This suggests the absence of an association between BMI and IMT may be explained by a lack of exposure to obesity in this cohort. Consistent with previous studies, tobacco use did not associate with IMT in the present study. A lack of association between IMT and smoking may be due to the relatively recent onset of smoking.^36^

There was no association between IMT with BP and impaired glycemia. High IMT may results from development of fatty streaks within the intima of the blood vessel wall as part of the atherosclerotic process. In addition, vascular smooth cell hypertrophy within the media may also explain increased intima‐media thickness. Smooth muscle cell hypertrophy is primarily caused by elevated BP^41^ and could explain the observed association between IMT and BP which has previously been reported.^17^, ^34^, ^36^, ^37^ In the present study, a lack of association between IMT and BP suggests that variation in IMT may primarily be due to intimal changes as part of the atherosclerotic process rather than vascular remodeling due to raised BP. The present study found no association between measures of impaired glycemia and IMT. It is well established that individuals with diabetes have a higher risk of cardiovascular events compared to non‐diabetic individuals. However, the association between measures of impaired glycemia in non‐diabetic patients to cardiovascular events is inconsistent^42^ and in line with the findings of the present study.

### 4.1 Strengths and limitations

The present study has several methodological strengths which should be noted. Firstly, to the best of our knowledge this is the first study to investigating the association between PWV and IMT to traditional cardiovascular risk factors in a cohort of young individuals from a transitioning rural community in Southern India which are high‐risk areas for cardiovascular disease. Secondly, the outcome variables were measured using gold‐standard techniques for assessing aortic stiffness and carotid atherosclerosis which are important intermediate measures of vascular disease that are predictive of future cardiovascular disease.

There are also several limitations to the present study which should be noted. The cross‐sectional nature of the present study does not allow determination of the direction of association. However, current understanding makes it highly unlikely that PWV or IMT may lead to changes in traditional risk factors. PWV and IMT were measured in triplicate with operator blinded to exposure variables (other than BMI) reducing the impact of observer bias. In addition, IMT measurement was confined to the carotid artery and may not represent the extent of atherosclerosis in other vascular beds. This non‐differential misclassification could bias the results toward the null. Additionally, exposure variables were measured only at one time point and may not represent accumulative exposure, if this single measurement underestimated accumulative exposure this would reduce the magnitude of the estimated effect for that exposure and vice versa. From the data available, it is not clear in what direction the bias would occur. We did not measure alcohol consumption in units per week which would be a more accurate measure. However, we feel that this is unlikely to impact our main conclusions as alcohol consumption was low with most individuals (85%) drinking only 1‐2 times a month (of these 49% were completely abstinent).

## 5 CONCLUSION

In the present cross‐sectional study including male and female young adults from Southern India we found strong evidence of an (a) positive association between PWV and BP which accounted for 37% of the variability in PWV and (b) negative association between IMT and HDL‐cholesterol which accounted for 2% of the variability in IMT.

## CONFLICT OF INTEREST

None.

## AUTHOR CONTRIBUTIONS

MC contributed to design of work, analysis and interpretation of data, and drafting of manuscript. RS contributed to design of work and acquisition of data. BK contributed to design of work and acquisition of data and critical appraisal of manuscript. SK contributed to important intellectual content and critical appraisal of manuscript. DN contributed to design of work, interpretation of data, and critical appraisal of manuscript.
