# Supplementary material for: Association of pulse wave velocity and intima‐media thickness with cardiovascular risk factors in young adults
Source: J Clin Hypertens (Greenwich). 2020 Jan 19;22(2):174–84. doi: 10.1111/jch.13812 (PMC7064924; doi:10.1111/jch.13812)
Supplement: Supplementary file 1 [file JCH-22-174-s001.docx]

# SUPPLEMENTAL MATERIAL

## Participant Characteristics by Missing Data

To assess the generalisability of the individuals included in the final regression model for IMT and PWV, participants with complete data and those with missing data were compared Table 1 and 2). For IMT, there was no evidence that individuals with complete data differed to those with missing data for any exposure variable (Table.1). For PWV, there was evidence that those that had missing data were older, more likely to be female, had higher HR, drank less and had a higher total cholesterol, HDL-cholesterol and HOMA-IR compared to those with complete data (Table.2).

**Sensitivity Analysis**

Two sensitivity analyses were conducted. Firstly, to determine the impact of outliers (defined as extreme values that clearly deviated from the distribution) on the final multivariable regression model. Variables where outliers were visible included total cholesterol (3 outliers were removed), heart rate (one data point was removed) and glucose (5 outliers were removed). The final multivariable regression analysis was repeated without the outliers to assess their impact on the data analysis. Secondly, IMT had a very slight right skew which was not improved by log transformation so the final multivariable regression model was restricted to those to the left of the IMT distribution tail (>0.9mm) to assess the consistency of the results.

None of the sensitivity analysis provided results that differed appreciably from the original analysis and the findings were robust in regard to a) removing outliers from the final regression models (Table.3 and .4) b) restricting analysis to those with an IMT value <0.90mm (Table.5). Restricting analysis to participants with and IMT measure <0.90mm did not alter and conclusions from the multivariable regression analysis between the association of IMT to HDL. However, evidence for an association between IMT and BMI became weaker (β=0.02 for 10 unit increase in BMI; 95%CI:0.00.0.04, P=0.174; Table.5).

**Table 1.** Characteristics of participants by availability of IMT data.

| **Characteristics** | **With IMT**  **(N=1362)** | **No IMT**  **(N=78)** | **P value** |
| --- | --- | --- | --- |
| **Age (years)** | 20.9 ± 1.2 | 20.8 ± 1.3 | 0.583 |
| **Male Sex (n [%])** | 815 [59.8] | 43 [60.6] | 0.903 |
| **BMI (kg/m^2^)** | 19.6 ± 2.8 | 19.1 ± 3.0 | 0.139 |
| **HR (bpm)** | 76.1 ± 14.3 | 76.0 ± 15.0 | 0.926 |
| **MAP (mm Hg)** | 84.0 ± 7.5 | 84.5 ± 7.7 | 0.551 |
| **Tobacco *** |  |  |  |
| **1. Never (n[%])** | 1177 [86.4] | 62 [81.6] | 0.329 |
| **2. Former (n[%])** | 10 [0.7] | 2 [2.6] |  |
| **3. Current (n[%])** | 175 [12.9] | 12 [15.8] |  |
| **Alcohol **** |  |  |  |
| **1. Daily/most days (n, [%] )** | 29 [2.13] | 1 [1.92] | 0.637 |
| **2. Weekends only (n[%])** | 190 [14.0] | 4 [7.69] |  |
| **3. 1-2 times a month (n[%])** | 253 [18.6] | 18 [34.6] |  |
| **4. Special occasions (n[%])** | 224 [16.5] | 8 [15.38] |  |
| **5. Never (n[%])** | 666 [48.9] | 21 [40.38] |  |
| **Total Cholesterol (mg/dL)** | 154.0 ± 34.5 | 156.3 ± 33.7 | 0.563 |
| **HDL-Cholesterol (mg/dl)** | 39.5 ± 7.5 | 40.2 ± 8.4 | 0.425 |
| **Log Triglycerides** | 4.5 ± 0.4 | 4.5 ± 0.4 | 0.705 |
| **Glucose (mg/dl)** | 86.4 ± 9.6 | 86.6 ± 8.9 | 0.866 |

*Smoking or chewing tobacco status, former user = ceased use >6 months ago; current user = used in the last 6 months; ** the highest frequency for either local spirits, branded spirit, wine or beer was used for analysis; $ Chi squared test for trend. BMI=body mass index; HR=heart rate, SBP=systolic blood pressure; DBP=diastolic blood pressure; MAP=mean arterial blood pressure; HDL=high density lipoprotein; PWV=pulse wave velocity; cIMT=carotid intima-media thickness.

**Table 2.** Characteristics of participants by availability of PWV data.

| **Characteristics** | **With PWV (N=1400)** | **No PWV (N=40)** | **P value** |
| --- | --- | --- | --- |
| **Age (years)** | 20.8 ± 1.2 | 21.2 ± 1.4 | 0.055 |
| **Male Sex (n [%])** | 848 [60.6] | 10 [30.3] | <0.001 |
| **BMI (kg/m^2^)** | 19.5 ± 2.8 | 19.7 ± 3.0 | 0.634 |
| **HR (bpm)** | 75.9 ± 14.3 | 83.8 ± 16.2 | 0.003 |
| **MAP (mm Hg)** | 84.0 ± 7.5 | 83.8 ± 7.5 | 0.890 |
| **Tobacco *** |  |  |  |
| **1. Never (n[%])** | 1204 [86.0] | 35 [92.1] | 0.307 |
| **2. Former (n[%])** | 12 [0.9] | 0 [0] |  |
| **3. Current (n[%])** | 184 [13.1] | 3 [7.8] |  |
| **Alcohol **** |  |  |  |
| **1. Daily/most days (n, [%] )** | 30 [2.14] | 0 [0] | 0.007 |
| **2. Weekends only (n[%])** | 192 [13.7] | 2 [5.3] |  |
| **3. 1-2 times a month (n[%])** | 271 [19.4] | 6 [15.8] |  |
| **4. Special occasions (n[%])** | 232 [16.6] | 2 [5.3] |  |
| **5. Never (n[%])** | 675 [48.2] | 28 [73.7] |  |
| **Total Cholesterol (mg/dL)** | 153.8 ± 34.3 | 165.2 ± 41.1 | 0.045 |
| **HDL-Cholesterol (mg/dl)** | 39.4 ± 7.5 | 41.8 ± 8.9 | 0.056 |
| **Log Triglycerides** | 4.5 ± 0.4 | 4.6 ± 0.5 | 0.101 |
| **Glucose (mg/dl)** | 86.5 ± 9.5 | 84.5 ± 10.8 | 0.206 |

*Smoking or chewing tobacco status, former user = ceased use >6 months ago; current user = used in the last 6 months; ** the highest frequency for either local spirits, branded spirit, wine or beer was used for analysis; $ Chi squared test for trend. BMI=body mass index; HR=heart rate, SBP=systolic blood pressure; DBP=diastolic blood pressure; MAP=mean arterial blood pressure; HDL=high density lipoprotein; PWV=pulse wave velocity; cIMT=carotid intima-media thickness.

**Table 3.** Final multivariate regression model of the association between PWV and cardiovascular risk factors excluding outliers.

|  |  | **β** | **95% Confidence Interval** | **P** | **R^2^** |
| --- | --- | --- | --- | --- | --- |
|  |  |  |  |  | 0.38 |
| MAP (mm Hg/10) |  | 0.45 | 0.41 to 0.49 | <0.001 |  |
| Glucose (mg/dl*10) |  | 0.02 | -0.01 to 0.06 | 0.234 |  |
| HOMA-IR |  | -0.01 | -0.06 to 0.04 | 0.674 |  |
| BMI (kg/m^2^) |  | 0.01 | <0.01 to 0.02 | 0.045 |  |

Model is adjusted for age, gender, village clustering, total cholesterol, HDL-cholesterol, triglycerides, smoking and alcohol consumption. BMI=body mass index; MAP=mean arterial blood pressure; HOMA-IR = homeostatic model assessment of insulin resistance.

**Table 4.** Final multivariable regression model of the association between cIMT and cardiovascular risk factors excluding outliers.

|  |  | **β** | **95% Confidence Interval** | **P** | **R^2^** |
| --- | --- | --- | --- | --- | --- |
|  |  |  |  |  | 0.02 |
| **Total cholesterol (mg/dL*10)** |  | 0.00 | -0.02 to 0.02 | 0.680 |  |
| **HDL-cholesterol (mg/dL*10)** |  | -0.10 | -0.18 to 0.02 | 0.013 |  |
| **BMI (kg/m^2^)** |  | 0.02 | 0.00 to 0.05 | 0.088 |  |
| **Tobacco use^#^** |  |  |  | 0.220^$^ |  |
| ***Never*** |  | Ref |  |  |  |
| ***Former*** |  | -0.28 | -0.75 to 0.20 |  |  |
| ***Current*** |  | 0.15 | -0.05 to 0.34 |  |  |
| **Triglycerides (mg/dL*10)** |  | -0.04 | -0.24 to 0.15 | 0.638 |  |

Model adjusted for age, gender, alcohol, mean arterial pressure, glucose, homeostatic model assessment of insulin resistance and village clusters #Smoking or chewing tobacco status, former user = ceased use >6 months ago; current user = used in the last 6 months; $=P-value for all categories; BMI=body mass index; HDL=high density lipoprotein.

**Table 5.** Final multivariable regression model of the association between cIMT and cardiovascular risk factor restricted to participants with and IMT under 0.9mm.

|  |  | **β** | **95% Confidence Interval** | **P** | **R^2^** |
| --- | --- | --- | --- | --- | --- |
|  |  |  |  |  | 0.02 |
| **Total cholesterol (mg/dL*10)** |  | 0.00 | -0.02 to 0.02 | 0.925 |  |
| **HDL-cholesterol (mg/dL*10)** |  | -0.10 | -0.15 to 0.02 | 0.013 |  |
| **BMI (kg/m^2^)** |  | 0.02 | 0.00 to 0.04 | 0.175 |  |
| **Tobacco use^#^** |  |  |  | 0.224^$^ |  |
| ***Never*** |  | Ref |  |  |  |
| ***Former*** |  | -0.21 | -0.66 to 0.25 |  |  |
| ***Current*** |  | 0.18 | -0.05 to 0.40 |  |  |
| **Triglycerides (mg/dL*10)** |  | -0.02 | -0.15 to 0.18 | 0.854 |  |

Model adjusted for age, gender, alcohol, mean arterial pressure, glucose, homeostatic model assessment of insulin resistance and village clusters #Smoking or chewing tobacco status, former user = ceased use >6 months ago; current user = used in the last 6 months; $=P-value for all categories; BMI=body mass index; HDL=high density lipoprotein.
